# Color Masking Ability of Guided Enamel Regeneration with a Novel Self-Assembling Peptide and Resin Infiltration on Artificial Enamel Lesions Under Various Challenges: An In Vitro Spectrophotometric Analysis

**DOI:** 10.3390/biomimetics9120764

**Published:** 2024-12-16

**Authors:** Nassreen Albar, Syed Nahid Basheer, Mohammed M. Al Moaleem, Sana Ageel, Rehab Abbas, Rafaa Hakami, Arwa Daghrery, Mohammed Sawady, Syed Wali Peeran, Thilla Sekar Vinothkumar, Bassam Zidane

**Affiliations:** 1 Department of Restorative Dental Sciences, College of Dentistry, Jazan University, Jazan 45142, Saudi Arabia; nalbar01@gmail.com (N.A.); ardaghrery@jazanu.edu.sa (A.D.); vinothkumar_ts@yahoo.com (T.S.V.); 2 Department of Prosthetic Dental Sciences, College of Dentistry, Jazan University, Jazan 45142, Saudi Arabia; malmoaleem@jazanu.edu.sa; 3 Interns Affairs Unit, College of Dentistry, Jazan University, Jazan 45142, Saudi Arabia; ageelsana950@gmail.com (S.A.); alabbas.rehab@gmail.com (R.A.); rafaa.hakami@gmail.com (R.H.); 4 Department of Preventive Dental Sciences, College of Dentistry, Jazan University, Jazan 45142, Saudi Arabia; mesawady@jazanu.edu.sa (M.S.); doctorsyedwali@gmail.com (S.W.P.); 5 Restorative Dentistry Department Sciences, Faculty of Dentistry, King Abdulaziz University, Jeddah 21589, Saudi Arabia; bzidane@kau.edu.sa

**Keywords:** guided enamel regeneration, self-assembling peptide, curodont repair fluoride plus, resin infiltration, initial carious lesions, artificial enamel lesions, white spot lesions

## Abstract

The color masking ability of resin infiltration (RI) and curodont repair fluoride plus–self-assembling peptide (CRFP-SAP) was investigated under various simulated oral challenging conditions. Sixty-four extracted caries-free human canines were randomly divided into two groups: Group 1 (RI) and Group 2 (CRFP-SAP). The baseline color values of samples were recorded using a spectrophotometer (VITA Easyshade^®^ Advance 4.0 VITA Zahnfabrik, Bad Sackingen, Germany). The samples were stored in a demineralization solution for 4 days to induce artificial enamel lesions (AELs). The AELs of Groups I and II were treated with RI (Icon, DMG, Hamburg, Germany) and CRFP-SAP (vVARDIS, Zug, Switzerland), respectively, followed by color measurements. Each group was subjected to challenges such as remineralization, pH cycling, staining, and thermocycling, followed by color measurements. The difference between the mean ∆E (color difference value) of sound enamel and both treatment groups was less than 3.7 1-week post treatment. Meanwhile, the difference between the mean ∆E of RI-treated samples and all kinds of challenges was more than 3.7, while for the CRFP-SAP-treated samples, it was less than 3.7 for all kinds of challenges, except for the thermocycling, for which the mean ∆E difference was 4.3. RI and CRFP-SAP treatments were effective in masking the discoloration caused by AELs. However, the color was not stable for RI-treated samples, whereas it was stable for CRFP-SAP-treated samples under all challenges, except for thermocycling.

## 1. Introduction

The color of dental enamel can be either yellow or milky white and is influenced by a multitude of factors, including its structural properties, external treatments, and environmental interactions. The inherent properties of the tooth on which the perception of the color depends include its hue, value, and chroma. One primary factor influencing the perception of tooth color is the thickness of enamel and the interplay between enamel and underlying dentin [1,2]. Also, different individuals, depending on many factors like their experience, gender, etc., may perceive color differently [3]. White spot lesions on enamel are a common dental condition that appear as opaque white spots on the tooth surface. Various types of enamel pathologies, like Amelogenesis Imperfecta, Molar Incisor Hypomineralization (MIH), and developmental enamel hypoplasia, can present as white spot lesions. However, one of the most common etiologies of white spot lesions is initial carious lesions. The primary mechanism in the formation of these lesions involves the release of acidic byproducts of cariogenic bacteria in dental plaque, which lowers the pH in the oral cavity, leading to the dissolution of hydroxyapatite crystals in the enamel and resulting in the characteristic white spot appearance due to subsurface porosity beneath an intact surface layer [4,5].

This optical phenomenon is accentuated when the teeth are dry, as the differing refractive indices of air, water, and enamel enhance the visibility of the lesions [6]. Although the remineralization of WSLs is possible within the first six months after demineralization, it is a slow process that is only effective in the outermost 30 microns of enamel [7,8]. As a result, the lesions do not fully resolve and remain visible to the human eye. These white spot lesions (WSLs) are not only clinically unaesthetic but also if left untreated, a greater amount of the tooth structure may be destroyed. Traditionally, WSLs have been treated by applying remineralizing agents like 5% fluoride varnish and CPP-ACP compounds. However, because of its inability to penetrate deep inside the lesions, these agents do not mask the opaque WSLs [8,9].

Various new strategies and materials are being developed and investigated in restorative dentistry to restore carious lesions in order to enhance adhesion, microhardness, reduce viscosity, and include biomimetic properties like self-repairing ability [10,11,12]. Recent materials like bioactive glass, chitosan, and agarose showed some enhanced lesion remineralization. However, its color masking ability and its ability to withstand the dynamic pH variations in the oral cavity need further investigation [13,14]. Studies indicate that theobromine shows some potential for remineralization, although it is less effective than fluoride and other agents like tricalcium phosphate (TCP) [15]. Another natural agent, namely liquorice, has also demonstrated remineralizing properties due to its bioactive compounds. However, other physicochemical properties have to be investigated [16]. The effectiveness of hydroxyapatite in preventing dental caries has been supported by various studies, indicating its potential as a viable alternative or adjunct to fluoride treatments. However, more research is needed to confirm its clinical effectiveness, including its color masking ability and ability to arrest the dental caries [17].

One of the recent treatment options for these lesions is the resin infiltration (RI) technique. When the lesions are infiltrated with a low-viscosity resin, which has a refractive index similar to enamel, there is a change in the light scattering phenomenon, and as a result, the discoloration is masked [18,19]. RI was introduced in 2009, and ICON (DMG, Hamburg, Germany) is the only patented RI product on the market. However, the RI treatment has some drawbacks, such as a lack of remineralization potential and the reappearance of white spots under aging conditions [20].

Self-assembling peptides (SAPs) are biomimetic substances that have been recently used to repair bones, cartilages, the central nervous system, and the cardiovascular system [21,22,23]. The curodont repair (vVARDIS, Switzerland) self-assembling peptide is a biomimetic material that was introduced in 2016 to treat initial carious lesions. When applied to initial carious lesions, it provides a scaffold to attract phosphates and calcium, and has the potential to regenerate lost enamel. It was found that curodont repair maintained high mechanical strength, even at depths of 125 μm, outperforming fluoride-based agents in this regard [24]. This characteristic is particularly important as it suggests that the remineralized enamel can withstand the mechanical stresses of normal oral function better than enamel treated with traditional agents. Furthermore, curodont repair’s mechanism of action involves the self-assembly of peptides that mimic natural enamel proteins, promoting the formation of hydroxyapatite crystals in a manner akin to natural enamel development [25]. This biomimetic approach not only aids in remineralization but also aligns with the body’s natural processes, potentially leading to more stable and effective outcomes compared to inorganic remineralizing agents [26].

In 2019, curodo repair (vVARDIS, Switzerland) was improved by adding 500 ppm of sodium fluoride to the existing formulation, and a new product, curodont repair flouride plus (vVARDIS, Switzerland), was introduced to the market. The findings of systematic reviews and trials related to the use of SAPs are not consistent, and there are not enough reported studies specifically for the curodont repair flouride plus–self-assembling peptide (CRFP-SAP) [27,28]. Several physicochemical properties of SAPs need to be investigated, one of the most important of which is its color masking ability under various dynamic conditions [29,30,31]. Spectrophotometers have the ability to accurately record the color changes and express numerically [32,33]. Hence, this study aimed to evaluate and compare the effects of RI and CRFP-SAP on the color masking ability of artificial enamel lesions (AELs) under various simulated oral conditions using a spectrophotometer. The null hypothesis adopted in the current study is that the color masking ability of CRFP-SAP will not be better than RI on artificially induced enamel lesions in vitro.

## 2. Materials and Methods

### 2.1. Sample Preparation

**Figure 1:**
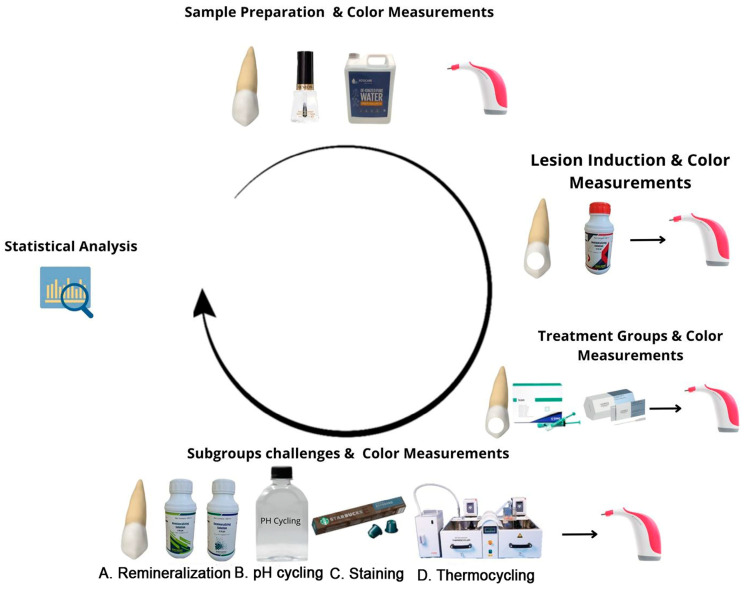
Schematic representation of stages of experiment.

The scientific research committee of the College of Dentistry, Jazan University, approved the use of 64 extracted non-carious human anterior canine teeth for the experiment (Ref No: CODJU-23141). The sample size of 64 teeth used in the present study was based on a power calculation to ensure adequate statistical power for detecting meaningful differences in color change between groups and subgroups. Based on a preliminary analysis and review of previous studies in similar contexts, it was suggested that a minimum sample size of 32 per group would provide sufficient power (80%) to detect a clinically significant color difference (ΔE ≥ 3.7), with a confidence level of 95% and an expected effect size of 0.8. These were further divided into 4 subgroups (*n* = 8 per subgroup) to assess color stability under various simulated oral challenges (remineralization, pH cycling, staining, and thermocycling). Following the standards set by the Centre for Disease Control and Prevention, the teeth were cleaned and sterilized [34]. All teeth were stored in pure deionized water (Eco Care LLC, Jeddah, Saudi Arabia) between different stages of the experiment (Figure 1). The root part of each tooth was put into a cylinder-shaped vinyl polysiloxane base (Putty Premium, Spident, Republic of Korea) for handling. A 4 mm diameter polyvinyl stencil was placed in the area of interest (AOI), which was at the center of the buccal surface of the crown of the teeth, The rest of the area that was visible was covered with acid-resistant nail paint (Revlon, NY, USA). Once the acid-resistant nail polish had dried, the polyvinyl stencil was removed from the AOI, and the samples were ready for the experiment. The samples were divided into two equal groups: the RI group (G1, *n* = 32) and the CRFP-SAP group (G2, *n* = 32). The color of the AOI of the sound samples from both G1 and G2 was recorded and considered the baseline color measurement.

### 2.2. Color Measurement Technique

A spectrophotometer (VITA Easyshade^®^ Advance 4.0 VITA Zahnfabrik, Bad Sackingen, Germany) was used to take digital measurements of color. For measuring color changes, the Commission Internationale de l’Eclairage (CIE) set global rules. These rules were followed when measuring color changes. In this color notation scheme, L represents lightness, a denotes the red–green coordinate, and b signifies the yellow–blue coordinate. Each time the spectrophotometer was switched on, it was calibrated before recording measurements. All color measurements were conducted under standardized lighting conditions. A daylight LED bulb with a color temperature of 5000 K was employed to calibrate the room illumination. A white background was used to measure the color during all stages of the experiment. To avoid variability, color measurements were recorded by the same investigator. The spectrophotometer’s tip measured 4 mm in diameter and was consistently maintained in a vertical orientation while resting on the specimen’s surface, which likewise had a diameter of 4 mm. The ∆E (color difference value), which is the color difference value between the point of measurement and the closest vita shade, was recorded three times for each sample, and the average was considered the final measurement. The mean ∆E of all samples of the treatment groups and subgroups was calculated. The change in color after the induction of artificial enamel lesions was determined by the difference between the mean ΔE of sound enamel and mean ΔE of the artificial enamel lesion. Similarly, the change in color after treatments was determined by calculating the difference between the mean ΔE of sound enamel and mean ΔE of the respective treatment group. Conversely, the change in color in different subgroup challenges was determined by calculating the difference between the ΔE of the treatment groups and ΔE of the respective subgroup challenges.

### 2.3. Artificial Enamel Lesion Induction

Once the baseline color measurements of the sound samples were recorded, the AELs were induced in the AOI by submerging the coronal segment of each tooth sample in 16 mL of a daily renewed demineralization solution containing 2.2 mM Calcium chloride and 2.2 mM potassium dihydrogen orthophosphate dehydrate in 0.05 M Acetic Acid. Next, 1 M potassium hydroxide was used drop by drop to adjust the pH to 4.4 and placed in an incubator at 37 ℃ for a period of 96 h to provide gentle agitation [35]. After being taken out of the demineralizing solution, the samples were carefully cleaned with deionized water. Using a spectrophotometer, color measurements were taken after 5 s of drying with an airway syringe. These were then compared to the baseline color measurements of the corresponding groups.

### 2.4. Treatment Groups

**Figure 2:**
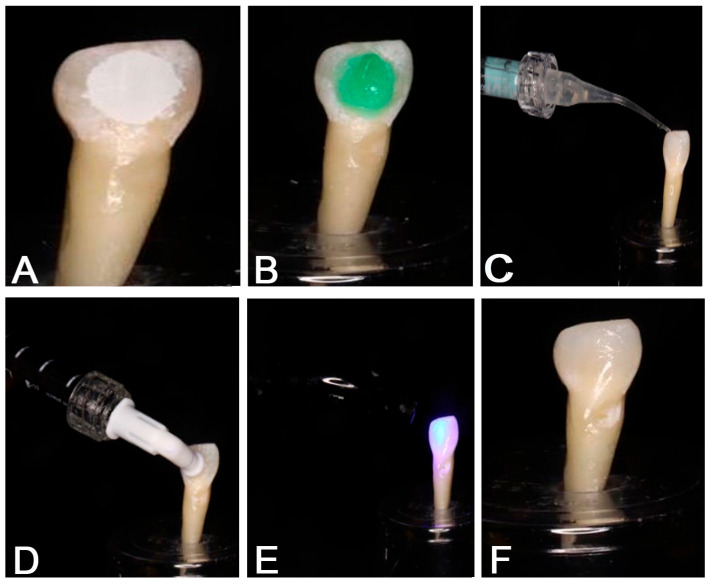
Resin infiltration application. (**A**) Artificial enamel lesions. (**B**) Icon-Etch application. (**C**) Icon-Dry application. (**D**) Icon-Resin infiltrant application, (**E**) Light Curing. (**F**) Post treatment.

Samples from Group 1 (RI group) were retrieved from deionized water, ensuring that all samples were sufficiently dry. Group 1 samples were subjected to RI. Following the manufacturer’s directions, the treatment was carried out. An ample amount of HCL acid gel etchant (Icon-Etch, DMG) was applied on the AELs using a vestibular tip attached to an etchant syringe. Occasionally, the tip was moved to the lesion to keep the etchant active for 2 min. The etchant was rinsed for 30 s with deionized water and dried with oil and water-free air. The AEL was then wetted with 99% ethanol (Icon-Dry; DMG) for 30 s to determine whether the discoloration had disappeared. If the discoloration did not disappear upon the application of Icon Dry, then the etching step was repeated. After confirming the masking of discoloration, oil- and water-free air was applied to the lesion to remove the Icon Dry. The vestibular tip was fixed to an Icon Infiltrant (Icon; DMG) syringe, and a sufficient amount of resin methacrylate infiltrant was applied to the AEL. Occasionally, the tip was moved to the lesion to allow the resin to infiltrate for 3 min. During this process, care was taken to avoid light exposure and to prevent the premature setting of the resin infiltrant. After 3 min, a LED light-curing unit (3M-ESPE, St. Paul, MN, USA) was used to facilitate the setting of the resin infiltrant for forty seconds. The infiltrate was re-applied for 60 s and light-cured for forty seconds (Figure 2).

**Figure 3:**
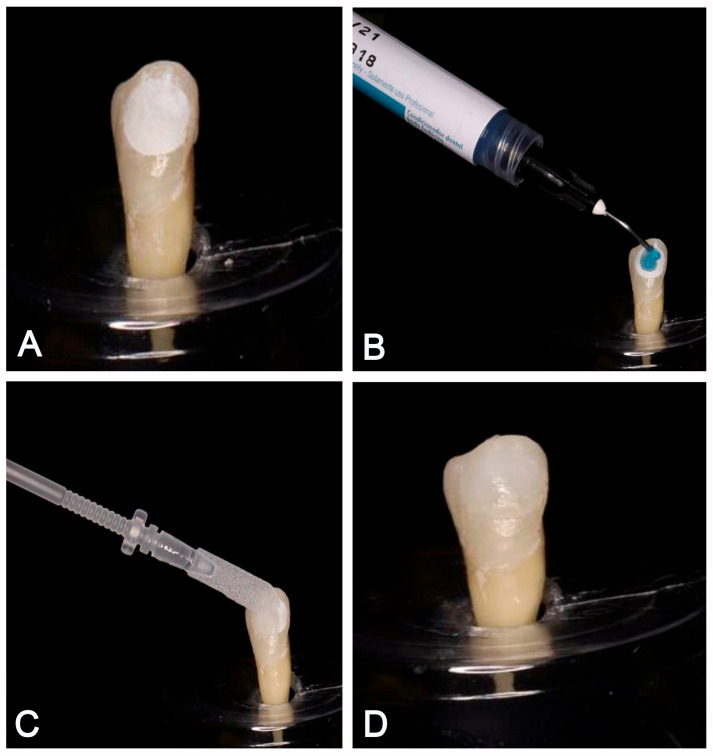
CRFP-SAP application. (**A**) Artificial enamel lesions. (**B**) Phosphoric acid gel application. (**C**) CRFP-SAP application. (**D**) Post treatment.

Samples from Group 2 (CRFP-SAP group) were retrieved from deionized water to ensure that they were sufficiently dry. Following the manufacturer’s directions, the treatment was carried out. Phosphoric acid (35%) was applied to the AEL of each sample for 20 s, followed by a thorough rinsing with deionized water. Damp cotton pellets were used to remove excess water and maintain a moist field. The CRFP-SAP powder was activated by removing the clip and plunging the sponge into a liquid reservoir. The sponge was soaked in the liquid for 10 s, retrieved, and placed on AEL under pressure for 5 min This process allows the CRFP-SAP to undergo a hierarchical self-assembly process, creating a fibrillar scaffold which will facilitate the deposition of calcium and phosphate ions from the saliva to penetrate and fill the porosities in demineralized enamel [36,37] (Figure 3).

To simulate the normal oral conditions, each sample from both treatment groups was stored for 1 week in 16 mL of daily renewed artificial saliva containing 7.5 pH and containing 4.29 mM of NaCl, 3.90 mM of Na_3_ PO_4_, 1.1 mM of CaCl_2_, 17.98 mM of KCl, 0.05 mM of H_2_SO_4_, 0.08 mM of MgCl_2_, and 3.27 mM of NaHCO_3_ [38]. After one week, the treated surface was polished by using only fine (light orange) and ultrafine (yellow) polishing disks (Sof-Lex, 3M, St. Paul, MN, USA), along with a low-speed handpiece with a speed set at 1200 RPM. Each disk was used for 15 s intermittently and unidirectional. The color measurements of the Group 1 RI-treated samples were recorded and compared with those of the respective Group 1 sound samples. Similarly, the color measurements of the Group 2 CRFP-SAP-treated samples were recorded and compared with those of the respective Group 2 sound samples.

### 2.5. Subgroup Challenges

Both treatment groups, namely Group 1 (RI group) and Group 2 (CRFP-SAP group), were equally divided into four subgroups (*n* = 8) to evaluate the color stability under various simulated challenges that occurred in the oral cavity over time. The colors of the subgroup samples after various challenges were recorded and compared with those of the respective samples from both treatment groups.

#### 2.5.1. Subgroup 1: Remineralization

To simulate the oral conditions where protective factors override the pathological factors during caries balance, GC Tooth Mousse (GC America Inc., Alsip, IL, USA) was applied to samples from subgroup 1 of group 1 (RI-treated group) and subgroup 1 of group 2 (CRFP-SAP-treated group), following the manufacturer’s instructions. A pea-sized amount of GC Tooth Mousse was placed on a gloved finger and applied to the AOI of each sample. After 5 min, each sample was stored in 16 mL of remineralizing solution with pH 7.0 containing 150 mM KCl, 0.9 mM NaH_2_PO_4_∙2H_2_O, 1.5 mM Ca(NO_3_)_2_∙4H_2_O, 0.1 mol/L Tris buffer, and 0.03 ppm F. Samples were placed in the incubator, maintaining a temperature of 37 °C centigrade. The solution was refreshed after 3 days [39]. After seven days, the remineralized subgroup samples were removed from the incubator, and the samples’ color measurements were noted, and then compared to the corresponding samples from the treatment groups

#### 2.5.2. Subgroup 2: pH Cycling

To simulate the oral conditions where pathological and protective factors are in a cycle to balance caries, the demineralization–remineralization protocol was followed. Each sample from subgroup 2 of group 1 (RI-treated group) and subgroup 2 of group 2 (CRFP-SAP-treated group) was submerged in 16 mL of demineralizing solution (pH 4.7) containing 2.0 mM Ca(NO_3_)_2_∙4H_2_O, 2.0 mM NaH_2_PO_4_∙2H_2_O, 0.075 mM acetate buffer, and 0.02 ppm for 6 h, followed by 16 mL of remineralizing solution (pH 7.0) containing 150 mM KCl, 0.9 mM NaH_2_-PO_4_∙2H_2_O, 1.5 mM Ca(NO_3_)_2_∙4H_2_O, 1.5 mM Ca(NO_3_)_2_∙4H_2_O, 0.03 ppm F, and 0.1 mol/L Tris buffer for 18 h. This cycle was repeated for five days, with the specimens remaining in the remineralizing solution for two more days. Samples were placed in the incubator, maintaining a temperature of 37 °C Celsius. The solution was refreshed after 3 days [39]. After seven days, the pH-cycled subgroup samples were removed from the incubator, and the samples’ color measurements were noted, and then compared to the corresponding samples from the treatment groups.

#### 2.5.3. Subgroup 3: Staining

To simulate staining challenges in the oral cavity, each sample from subgroup 3 of group 1 (RI-treated group) and subgroup 3 of group 2 (CRFP-SAP-treated group) was submerged in 25 mL of staining solution made from espresso roasted coffee packed in a capsule (Nestlé S.A., Vaud, Switzerland). Coffee was brewed by setting a high pressure of 19-bar on a pump capsule machine (Nespresso, De’Longhi, Treviso, Italy). The staining solution was let to cool to 37 °C before submerging the samples. Everyday freshly brewed coffee solution was used for eight days [33]. At the end of the eighth day, samples were taken out of the solution, carefully washed with deionized water, and dried using absorbent paper, and the color of the samples was recorded and compared with the respective samples in the treatment groups.

#### 2.5.4. Subgroup 4: Thermocycling

To simulate the thermal challenges in the oral cavity, samples from subgroup 4 of Group 1 (RI-treated group) and subgroup 4 of Group 2 (CRFP-SAP-treated group) were submitted to a thermocycling unit (SD Mechatronik GmbH, Westerham, Germany) for 10,000 thermocycles in water temperatures between 5 °C and 55 °C for a period of 166.67 h, which simulates approximately 1 year of physiologic usage at a dwell time of 30 s in each bath [40]. The thermocycled subgroup samples were removed, and the color measurements of the samples were recorded and compared with those of the respective samples in the treatment groups.

### 2.6. Statistical Analysis

Descriptive and inferential statistical analyses were conducted in the present study using IBM SPSS Statistics 20.0 (IBM Corporation, Armonk, NY, USA). Microsoft Word and Excel were utilized to generate the tables (Tables 1–6). Paired sample *t*-tests were performed to determine the statistical significance of the study parameters on a continuous scale within and between the two groups. A *p*-value of ≤0.05 was considered statistically significant. The means and standard deviations (SDs) of the ΔE values for the sound samples, treatment group samples, and subgroup samples were recorded. In accordance with previous studies, a ΔE value difference of ≥3.7 between the compared groups and subgroups was considered a significant change in color [33,41].

## 3. Results

In Group 1, the mean ΔE difference between sound enamel (8.47 ± 3.78) and an artificial enamel lesion (4.13 ± 2.26) was 4.34, with a *p*-value of <0.001 (Table 1). Similarly, for Group 2, the mean ΔE difference between sound enamel (9.65 ± 4.36) and an artificial enamel lesion (4.62 ± 3.25) was 5.02, with a *p*-value of <0.001 (Table 1). Therefore, both groups showed a statistically significant difference (*p* ≤ 0.05) and clinically significant color change (mean difference ≥ 3.7).

**Table 1:** Color comparison between sound enamel and artificial enamel lesion.

Group 1 (RI)			95% CI		t	*p*-Value	Group 2 (CRFP-SAP)			95% CI		t	*p*-Value
Sound Enamel Mean ± SD	Artificial Enamel Lesion Mean ± SD	Mean ΔE Difference	Upper	Lower			Sound Enamel Mean ± SD	Artificial Enamel Lesion Mean ± SD	Mean ΔE Difference	Upper	Lower		
8.47 ± 3.78	4.13 ± 2.26	4.34 ^#^	5.927	2.753	5.578	<0.001 *	9.65 ± 4.36	4.62 ± 3.25	5.02 ^#^	6.204	3.845	8.689	<0.001 *

* Significant value ≤ 0.05; paired *t*-test; RI—resin infiltration; CRFP-SAP—curodont repair fluoride plus–self-assembling peptide; ^#^ mean difference ≥ 3.7 (significant color change).

The mean ΔE difference between sound enamel (8.47 ± 3.78) and RI-treated enamel (10.30 ± 2.26) was 1.828, with a *p*-value of 0.042 (Table 2). The mean ΔE difference between sound enamel (9.65 ± 4.36) and CRFP-SAP-treated enamel (8.55 ± 4.08) was 1.093, with a *p*-value of 0.041 (Table 2). Although both treatments exhibited a statistically significant difference (*p* ≤ 0.05), the mean color difference did not reach a clinically significant threshold of ≥3.7, indicating that there was no significant change in the color of both treatment groups from that of the sound samples.

**Table 2:** Color comparison between sound samples and treatment groups.

Group 1 (RI)			95% CI		t	*p*-Value	Group 2 (CRFP-SAP)			95% CI		t	*p*-Value
Sound Enamel Mean ± SD	Resin Infiltrated Mean ± SD	Mean ΔE Difference	Upper	Lower			Sound Enamel Mean ± SD	CRFP-SAP Mean ± SD	Mean ΔE Difference	Upper	Lower		
8.47 ± 3.78	10.30 ± 2.26	−1.828 ^#^	−0.069	−3.587	−2.120	0.042 *	9.65 ± 4.36	8.55 ± 4.08	1.093 ^#^	2.141	0.045	2.128	0.041 *

* Significant value ≤ 0.05; paired *t*-test; RI—resin infiltration; CRFP-SAP—curodont repair fluoride plus–self-assembling peptide; ^#^ mean difference ≥ 3.7 (significant color change).

The mean ΔE difference between the RI-treated samples (11.48 ± 3.99) and the RI-treated remineralized subgroup (7.13 ± 3.65) was 4.35, with a *p*-value of <0.001 (Table 3). The mean ΔE difference between the CRFP-SAP-treated samples (10.43 ± 5.11) and the CRFP-SAP-treated remineralized subgroup (7.72 ± 3.51) was 2.71, with a *p*-value of 0.035 (Table 3). Although both groups showed a statistically significant difference (*p* ≤ 0.05), only the RI-treated remineralized subgroup showed a clinically significant color change (mean difference ≥ 3.7).

**Table 3:** Color comparison between treatment group samples and remineralized subgroup samples.

Group 1 (RI)			95% CI		t	*p*-Value	Group 2 (CRFP-SAP)			95% CI		t	*p*-Value
RI-Treated Samples Mean ± SD	RI-Treated Remineralized Subgroup 1 Mean ± SD	Mean ΔE Difference	Upper	Lower			CRFP-SP-Treated Samples Mean ± SD	CRFP-SP -Treated Remineralized Subgroup 1 Mean ± SD	Mean ΔE Difference	Upper	Lower		
11.48 ± 3.99	7.13 ± 3.65	4.35 ^#^	5.903	2.796	6.623	<0.001 *	10.43 ± 5.11	7.72 ± 3.51	2.71 ^#^	5.170	0.254	2.609	0.035 *

* Significant value ≤ 0.05; paired *t*-test; RI—resin infiltration; CRFP-SAP—curodont repair fluoride plus–self-assembling peptide; ^#^ mean difference ≥ 3.7 (significant color change).

The mean ΔE difference between the RI-treated samples (11.81 ± 4.88) and the RI-treated pH-cycled subgroup (7.27 ± 4.32) was 4.53, with a *p*-value of 0.026 (Table 4). The mean ΔE difference between the CRFP-SAP-treated samples (6.91 ± 4.65) and the CRFP-SAP-treated pH-cycled subgroup (4.98 ± 3.68) was 1.92, with a *p*-value of 0.018 (Table 4). Although both groups showed a statistically significant difference (*p* ≤ 0.05), only the RI-treated pH-cycled subgroup showed a clinically significant color change (mean difference ≥ 3.7).

**Table 4:** Color comparison between treatment group samples and pH-cycled subgroup samples.

Group 1 (RI)			95% CI		t	*p*-Value	Group 2 (CRFP-SAP)			95% CI		t	*p*-Value
RI-Treated Samples Mean ± SD	RI-Treated pH-Cycled Subgroup 2 Mean ± SD	Mean ΔE Difference	Upper	Lower			CRFP-SP-Treated Samples Mean ± SD	CRFP-SP-Treated pH-Cycled Subgroup 2 Mean ± SD	Mean ΔE Difference	Upper	Lower		
11.81 ± 4.88	7.27 ± 4.32	4.53 ^#^	8.355	0.719	2.180	0.026 *	6.91 ± 4.65	4.98 ± 3.68	1.92 ^#^	3.413	0.436	3.058	0.018 *

* Significant value ≤ 0.05; paired *t*-test; RI—resin infiltration; CRFP-SAP—curodont repair fluoride plus–self-assembling peptide; ^#^ mean difference ≥ 3.7 (significant color change).

The mean ΔE difference between the RI-treated samples (9.58 ± 2.46) and the RI-treated stained subgroup samples (4.15 ± 2.35) was 5.43, with a *p*-value of <0.001 (Table 5). The mean ΔE difference between the CRFP-SAP-treated samples (10.46 ± 1.79) and CRFP-SAP-treated stained subgroup samples (12.77 ± 5.35) was 2.31, with a *p*-value of 0.216 (Table 5). Therefore, the RI-treated stained subgroup samples showed both a statistically significant difference (*p* ≤ 0.05) and clinically significant color change (mean difference ≥ 3.7). However, the CRFP-SAP-treated stained subgroup samples showed neither a statistically significant nor clinically significant color change.

**Table 5:** Color comparison between treatment group samples and stained subgroup samples.

Group 1 (RI)			95% CI		t	*p*-Value	Group 2 (CRFP-SAP)			95% CI		t	*p*-Value
RI-Treated Samples Mean ± SD	RI-Treated Stained Subgroup 3 Mean ± SD	Mean ΔE Difference	Upper	Lower			CRFP-SP-Treated Samples Mean ± SD	CRFP-SP-Treated Stained Subgroup 3 Mean ± SD	Mean ΔE Difference	Upper	Lower		
9.58 ± 2.46	4.15 ± 2.35	5.43 ^#^	7.284	3.590	6.962	<0.001 *	10.46 ± 1.79	12.77 ± 5.35	−2.31 ^#^	1.709	−6.334	−1.360	0.216

* Significant value ≤ 0.05; paired *t*-test; RI—resin infiltration; CRFP-SAP—curodont repair fluoride plus–self-assembling peptide; ^#^ mean difference ≥ 3.7 (significant color change).

The mean ΔE difference between the RI-treated samples (8.33 ± 3.91) and RI-treated thermocycled subgroup (14.52 ± 5.21) was 6.187, with a *p*-value of <0.001 (Table 6). The mean ΔE difference between the CRFP-SAP-treated samples and CRFP-SAP-treated thermocycled subgroup (10.71 ± 5.16) was 4.300, with a *p*-value of 0.05. Therefore, both the RI-treated thermocycled and CRFP-SAP-treated thermocycled subgroups had a statistically (*p* ≤ 0.05) and clinically significant color change (mean difference ≥ 3.7).

**Table 6:** Color comparison between treatment group samples and thermocycled subgroup samples.

Group 1 (RI)			95% CI		t	*p*-Value	Group 2 (CRFP-SAP)			95% CI		t	*p*-Value
RI-Treated Samples Mean ± SD	RI-Treated Thermocycled Subgroup Mean ± SD	Mean ΔE Difference	Upper	Lower			CRFP-SP-Treated Samples Mean ± SD	CRFP-SP-Treated Thermocycled Subgroup Mean ± SD	Mean ΔE Difference	Upper	Lower		
8.33 ± 3.91	14.52 ± 5.21	−6.187 ^#^	−3.840	−8.534	−6.234	<0.001 *	6.41 ± 2.49	10.71 ± 5.16	−4.300 ^#^	−1.765	−6.834	−4.012	0.05 *

* Significant value ≤ 0.05; paired *t*-test; RI—resin infiltration; CRFP-SAP—curodont repair fluoride plus–self-assembling peptide; ^#^ mean difference ≥ 3.7 (significant color change).

## 4. Discussion

**Figure 4:**
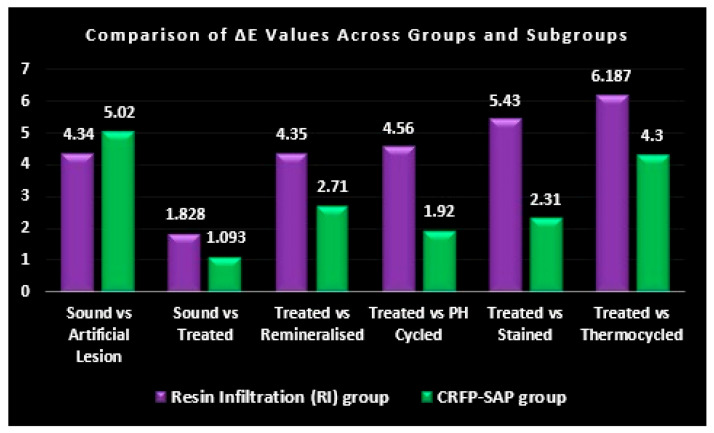
Comparison of mean color difference between RI (resin infiltration) and CRFP-SAP (curodont repair fluoride plus–self-assembling peptide).

The null hypothesis adopted in this study was rejected because the color masking ability of CRFP-SAP was better than RI on artificially induced enamel lesions under all challenging conditions. The VITA Easy Shade spectrophotometer was used to measure color in the current study, because studies have shown that instrumental color matching using the VITA Easy Shade spectrophotometer is more reliable and reproducible than visual assessments performed by clinicians, which can be influenced by lighting conditions and individual perception [42,43]. This device quantifies the spectral dispersion of light and translates it into three color values (L*, a*, and b*), as per the criteria established by the International Commission on Illumination. L* quantifies the level of color lightness in a specimen, with a scale from 0 to 100, where 0 denotes black and 100 denotes white. Conversely, a* and b* represent chromaticity without defined numerical constraints. A negative a* value indicates green, a positive value indicates red, a negative b* value indicates blue, and a positive value indicates yellow. The changes in the (L*, a*, b*) values are computed automatically in the spectrophotometer to give the ΔE value, which is the color difference value between the point of measurement and the closest vita shade value. The specific formula which is used by the spectrophotometer to obtain ΔE is the following: ΔE = √((ΔL*)^2^ + (Δa*)^2^ + (Δb*)^2^) [44,45,46]. ∆E was used in this study for statistical analysis purposes because it gives a value considering all three components of color, including value, chroma, and hue. Previous studies have shown that if the ∆E is less than 3.7, the human eye finds it challenging to detect any alteration in color [39,41]. Thus, in this study, when comparing between groups and subgroups, if the ∆E was more than or equal to 3.7, it was considered a clinically significant change in color (Figure 4).

Since the current study included procedures such as demineralization, remineralization, guided enamel regeneration, and pH cycling, it was important to prevent ion exchange between the experiments. Therefore, the samples were stored in deionized water between the experiments [47]. To target ion exchange only at the AOI (4 mm), the remaining exposed surface was covered with an acid-resistant nail polish (REVLON, New York, NY, USA) [39,48]. The volume of the demineralizing and remineralizing solutions was determined using a formula from a previous study, as follows: total volume = 2 mL solution /1 mm^2^ enamel area [49]. As the AOI was 4 mm^2^, each tooth was subjected to 16 mL of the solution. Using this protocol, researchers were able to produce subsurface lesions of 120–200 microns deep, which is close to the approximate depth of actual initial carious lesions, which is around 50 to 290 microns [35,50,51,52].

However, to validate the formation of AELs in this study, the color measurements of the AELs were recorded and compared with the baseline measurements of the sound samples. Comparison of the color differences between the sound and artificial enamel lesions showed significant differences in both groups (Table 1), with the mean differences exceeding the clinically significant threshold of 3.7. Thus, lesion formation was confirmed. In addition, a robust and detailed lesion assessment was performed by three examiners for each sample based on the International Caries Detection and Assessment System (ICDAS) criteria, which is one of the most accepted ways of detecting initial carious lesions [53]. All samples were given an ICDAS Score of 2 by all three examiners, which means that there was a distinct visual change in all samples.

The AELs of the G1 and G2 samples were treated with RI and CRFP-SAP, respectively, according to the manufacturer’s instructions. Since HCL acid was used to etch before the application of RI and phosphoric acid was used before the application of CRFP-SAP, it is expected that there will be differences in the thickness and characteristics of the hard surface layer of the enamel. However, this will not be the source for bias in the study because the aim of the study itself was to compare the effects of two different products with different protocols on the color masking ability of artificial enamel lesions. To simulate normal oral conditions after the treatment procedures, samples from both treatment groups were stored for 1 week in an artificial salivary solution that was close to the contents of saliva [38]. Finishing and polishing procedures in both of these treatments are critical, because the expected depth of the artificial lesions formed itself was around 120–200 micrometers. Any aggressive finishing and polishing may result in the removal of the material from the surface. Excessive material was removed with an explorer before the setting of the materials, precluding the necessity of finishing with the coarse grain polishing disks. Only flexible fine (light orange) and ultrafine (yellow) soflex polishing disks with smaller granularity were used for reasonable polishing. Each disk was used for 15 s intermittently and unidirectional. A similar polishing protocol demonstrated that there is a significant decrease in the average roughness of resin-infiltrated caries lesions in enamel [54]. The same protocol was followed for CRFP—SAP also, because no specific finishing protocol was found in the published literature nor was specified by the manufacturer.

The color measurements of the treated samples were recorded one week later because studies have shown that the maximum remineralizing potential of SAP is reached after one week. When the samples were kept in the remineralizing solution for 4 weeks, there was no significant increase in microhardness [55]. For the proper color measurement of RI, at least 2 days of time should be given for rehydration [56]. However, to remove bias, both groups of samples were kept in solution for 1 week. When the color comparison was performed between the sound samples and treatment groups, the mean differences did not reach a clinically significant threshold of 3.7 for either of the groups (Table 2), indicating that there was no significant change in the color in either of the treatment groups. Hence, both the RI and CRFP-SAP treatments were effective in masking the discoloration caused by AELs at 1-week post treatment. The findings of the current study regarding RI are in agreement with those of a previous systematic review concluding that RI treatment of white spot lesions is effective during the initial period of 3 months [20]. The findings of the present study are indirectly in agreement with those of a recent study in which CRFP-SAP (Credentis AG, Switzerland) showed promising results in remineralizing white spot lesions [57]. However, a previous study showed that the application of self-assembling peptides could neither mask the lesion nor inhibit lesion progression [58]. These different findings can be attributed to the use of CR-SAP in an earlier study and CRFP-SAP in the current study.

Each treatment group was equally divided into four subgroups: remineralization (*n* = 8), pH cycling (*n* = 8), staining (*n* = 8), and thermocycling (*n* = 8). To simulate an oral condition in which enhanced remineralization is provided in the mouth, each sample from both treatment subgroups was individually painted with GC Tooth Mousse, which contains phosphate and calcium, in a special milk-derived protein called casein phosphopeptide–amorphous calcium phosphate. When color was compared between the treatment group samples and their respective remineralized subgroup samples, both groups showed statistically significant color changes; however, only the RI group showed a clinically significant color change (mean difference ≥ 3.7) (Table 3). The results of the RI-treated remineralized samples in the present study indirectly agree with a previous study in which it was concluded that when RI is combined with remineralizing agents, there is no improvement in the efficiency of the treatment [59]. In contrast to RI-treated remineralized samples, CRFP-SAP-treated remineralized samples were effective in masking the discoloration resulting from AELs and are indirectly in agreement with a recent study on CR, which is close to the novel CRFP-SAP [55]. This might be because when SAP is applied to initial carious lesions, it creates a scaffold that attracts the phosphates and calcium present in the saliva and regenerates the enamel [60].

To simulate an oral condition when repeated cycles of demineralization and remineralization take place at the tooth surface, samples were subjected to a pH-cycling protocol wherein each sample from the subgroup was kept in demineralizing solution for 6 h and remineralizing solution for 18 h alternately before recording measurements. The pH-cycling protocol followed in this study was adopted from a previous study in which the average time for demineralization in the mouth was 6 h per day, whereas the average time for remineralization was 18 h per day [39,61]. When color differences were compared between the treatment group and the pH-cycled subgroup samples, it was found that the RI-treated pH-cycled subgroup had a clinically significant color change (mean difference ≥ 3.7) (Table 4). However, there was no clinically significant change in color (mean difference < 3.7) (Table 4) in the CRFP-SAP-treated pH-cycled subgroup samples. The results of the RI-treated pH-cycling samples in the current study are in agreement with previous studies using similar pH-cycling protocols, which showed decreased microhardness of the RI-treated lesions, resulting in the formation of distinct surface discoloration [48,62,63]. The results of the CRFP-SAP-treated pH-cycling samples in the present study indirectly agree with previous studies, and this might be because a net mineral gain in artificial white spot lesions was detected when the SAP-treated teeth were subjected to pH cycling [30,64].

To simulate the oral conditions of extrinsic staining, a staining protocol from a previous study was followed [33]. Coffee was used to stain the treated RI and CRFP-SAP samples to check whether the color was stable after the staining challenge. Studies have shown that immersing samples in coffee solution for 24 h corresponds to approximately one month of coffee consumption, and in the present study, samples were submerged in the staining solution for eight days, which corresponds to eight months of coffee consumption [48,65,66]. When color comparison was performed between the RI-treated samples and the respective RI-treated stained subgroup samples, it indicated both a statistically and clinically significant color change (mean difference ≥ 3.7) (Table 5). However, there was no statistically or clinically significant change in color in the CRFP-SAP-treated stained subgroup samples (mean difference < 3.7) (Table 5). The results of the RI-treated stained samples in the current study are in direct agreement with those of earlier studies using similar staining conditions that demonstrated a clinically significant surface discoloration [33]. In contrast, the CRFP-SAP-treated teeth showed color stability after 8 months of staining. No previous studies have directly compared CRFP-SAP staining results with those of the present study. However, another study showed that when SAPs are used on bleached enamel, the surface roughness can be recovered [67]. Therefore, reduced staining in SAP-treated teeth may be related to decreased surface roughness.

To simulate the variations in temperature in the mouth and evaluate its effects on RI- and CRFP-SAP-treated surfaces, the samples were subjected to thermocycling. The acceptable test for aging dental materials, according to ISO standards, is 500 thermocycles in water temperatures ranging from 5 °C to 55 °C [68]. But research shows that leakage grows with heat stress, and 10,000 thermocycles is about the same as a year of in vivo function [40]. Therefore, in this study, the samples were submitted to 10,000 thermocycles. When color was compared between the RI-treated group samples and the RI-treated thermocycled subgroup samples, it indicated both a statistically and clinically significant color change (mean difference ≥ 3.7) (Table 6). Similarly, when the CRFP-SAP-treated group samples and the CRFP-SAP-treated thermocycled subgroup samples were compared, it showed that there was a statistical and clinically significant change in color (mean difference ≥ 3.7) (Table 6). These results are consistent with a recent systematic review and other studies that concluded that the RI treatment of white spot lesions is effective during the initial period; however, over time, there is a significant change in color [20,33]. No studies have directly compared the ability of CRFP-SAPs to mask discoloration during thermocycling. However, it seems that although CRFP-SAP remineralizes the initial carious lesions, the quality of the enamel formed cannot replicate that of natural enamel. Hence, the color masking ability was unstable under the thermocycling challenge.

The absence of a statistically significant color change for CRFP-SAP-treated samples compared to RI can be attributed to differences in their treatment mechanisms. RI relies on the infiltration and masking of enamel lesions with resin, which directly modifies the optical properties of the enamel, leading to immediate and visible changes in color. In contrast, CRFP-SAP does not involve masking but focuses on the gradual remineralization process, which may result in less pronounced short-term optical changes. Thus, while CRFP-SAP shows significant potential for enamel repair, its effects on color may not be as immediate or dramatic as those achieved by RI. Further studies with extended observation periods may better capture the long-term benefits of CRFP-SAP.

Overall, it seems like CRFP-SAP has an edge over RI because of its self-assembling peptide which forms nanotapes and fibrillar scaffolds, facilitating the penetration of calcium and phosphates from saliva continuously in the demineralized porosities, which is vital in dynamic mouth conditions [36,37]. The fluoride component of the CRFP-SAP also has the potential to inhibit demineralization and promote the remineralization of enamel, which is lacking in RI [24,31,69]. Additionally, studies have shown that unlike RI, SAP can reduce the viability of Streptococcus mutans, which is a primary bacterium involved in dental caries [70].

### Limitations

In the current study, samples were submitted for various simulating challenges for a limited period of time: (remineralization = 1 week), (pH cycling = 1 week), (staining = 8 months), and (thermocycling = 1 year). Further experiments must be conducted over a longer period of time and color changes should be also observed for tea stains. Future studies should be conducted with scanning electron microscopy (SEM) and atomic force microscopy (AFM) to understand the relationship between color and the characteristics of CRFP and RI at macro and nano levels. However, the current study is the first to compare the effects of CRFP-SAP and RI on the color masking ability of AELs under various challenges, and the initial results of CRFP-SAP are promising.

## 5. Conclusions

Within the limitations of this study, under the selected conditions, it can be concluded that both the RI and CRFP-SAP treatments can effectively mask the discoloration of artificial enamel lesions in the first week of treatment. However, RI treatment could not mask the discoloration under simulated aging conditions, such as remineralization, pH cycling, staining, and thermocycling challenges, whereas CRFP-SAP treatment could mask the discoloration under all challenges, except the thermocycling challenge. Although CRFP-SAP could not mask the color under the thermocycling challenge, its color masking ability was relatively better than that of the RI treatment. Further in vitro and in vivo studies are recommended to investigate the long-term color stability of CRFP-SAP using digital image analysis integrated with artificial intelligence.

## Data Availability

The raw data supporting the conclusions of this article will be made available by the authors on request.
